# Deposition and Optical Characterization of Sputter Deposited p-Type Delafossite CuGaO_2_ Thin Films Using Cu_2_O and Ga_2_O_3_ Targets

**DOI:** 10.3390/ma17071609

**Published:** 2024-04-01

**Authors:** Akash Hari Bharath, Ashwin Kumar Saikumar, Kalpathy B. Sundaram

**Affiliations:** Department of Electrical and Computer Engineering, University of Central Florida, Orlando, FL 32826, USA

**Keywords:** transparent conducting oxides, TCO, CuGaO_2_, delafossite, p-type, RF Sputtering, XRD, XPS, SEM, optical transmission, optical bandgap

## Abstract

CuGaO_2_ thin films were deposited using the RF magnetron sputtering technique using Cu_2_O and Ga_2_O_3_ targets. The films were deposited at room temperature onto a quartz slide. The sputtering power of Cu_2_O remained constant at 50 W, while the sputtering power of Ga_2_O_3_ was systematically varied from 150 W to 200 W. The films were subsequently subjected to annealing at temperatures of 850 °C and 900 °C in a nitrogen atmosphere for a duration of 5 h. XRD analysis on films deposited with a Ga_2_O_3_ sputtering power of 175 W annealed at 900 °C revealed the development of nearly single-phase delafossite CuGaO_2_ thin films. SEM images of films annealed at 900 °C showed an increasing trend in grain size with a change in sputtering power level. Optical studies performed on the film revealed a transmission of 84.97% and indicated a band gap of approximately 3.27 eV. The film exhibited a refractive index of 2.5 within the wavelength range of 300 to 450 nm.

## 1. Introduction

Transparent conducting oxides (TCOs) represent a distinct category of compounds distinguished by their notable optical transparency in the visible spectrum, coupled with excellent electrical conductivity [1]. As a result of these characteristics, TCOs find extensive applications across various optical devices, including light-emitting diodes, solar cells, sensors, and displays [2,3,4,5]. P-type and n-type TCOs can be potentially used in touch panels in hospitals and smartphones [6]. In this family of p-type TCOs, CuCrO_2_ is known to have antibacterial properties. Further, these antibacterial properties may find applications in biomedical implants, as reported by Jabed et al. in metallic glass-based materials [7]. TCOs are achieved by doping metal oxides by adding more charge carriers while essentially maintaining their optical characteristics. Due to high conductivity, n-type TCOs are used for conducting electrode applications [8]. Extensive research has been conducted on n-type TCOs such as Sn-doped In_2_O_3_ (ITO) [9,10], Sc-doped ZnO [11,12], and Al-doped ZnO (AZO) [13,14,15,16]. Nonetheless, there exists a lack of research on p-type TCOs, impeding the progress of transparent electronics applications [17,18,19]. In the case of p-type TCOs, it is difficult to synthesize a film that has both good optical transparency and high electrical conductivity.

The challenge in achieving a p-type TCO is due to the deep localization of holes at the O-2p oxygen level. This can be explained by the fact that the valence orbits of metallic atoms are located at a much higher energy level than O-2p levels [20,21]. Studies show that metal oxides have far lower valance band levels than metals. Doping offers a potential solution to this issue [22]. Nonetheless, the existence of highly electronegative O_2_ ions hinders the mobility of holes within the crystal lattice. To mitigate holes inside the crystal lattice, the localized holes would require a high amount of energy to overcome the large barrier height [23]. This is one of the main reasons why p-type TCOs have low electrical conductivity [24,25]. Hosono et al. introduced the idea of chemical modulation of the valence band (CMVB) as a solution to this problem [20]. It was discovered that incorporating cations like Cu+ results in the formation of a strong covalent bond with oxygen ions. This is because the energy difference between O 2p^6^ and Cu 3d^10^ is very small and Cu 3d^10^ forms a strong covalent bond with the O 2p^6^. As a result, the energy level of O-2p increases, leading to a decrease in the coulombic attraction between O_2_ ions and the holes. This increases conductivity by allowing holes to flow freely across the crystal lattice [22]. The optical transparency would be preserved due to the closed shell nature of Cu 3d^10^ which prevents coloration [25].

Films synthesized using the CMVB concept are called delafossites. The cation used for this process could be Cu+ or Ag+. Earlier research shows that the film made with Ag+ cation has very high electrical resistivity (10^4^–10^6^ Ω-cm) [25]. This is due to the exceptionally low carrier mobility resulting from the unfavorable energy alignment between O-p and Ag-4d levels [26]. Delafossite compounds incorporating Cu+ cations are referred to as Cu-based delafossites. Copper-based delafossites are gaining a lot of attention due to their large bandgap [19,27]. Cu-based delafossite materials are represented by the chemical formula CuMO_2_, with Cu denoting the positively charged monovalent cation (Cu+), M representing trivalent cations such as Cr^3+^, Ga^3+^, and Y^3+^, and oxygen serving as the negatively charged divalent anion (O^2−^). CuGaO_2_ belongs to the Cu-based delafossite family and is being researched extensively because of its electrical, optical, and photovoltaic properties [28,29,30]. Additionally, they could also be used for maintaining energy through solar water splitting, fuel cells, battery devices, and electrolyzers [31]. It has a high bandgap along with demonstrating excellent optical transparency and high hole mobility [32,33,34]. Numerous methods have been used to deposit p-type CuGaO_2_ films. The sol-gel deposition process performed by Ehara et al. uses copper nitrate trihydrate and gallium nitrate n-hydrate dissolved in 2-methoxyethanol [35]. Yu et al. performed a hydrothermal process to obtain delafossite CuGaO_2_. In this process, the precursor was prepared by dissolving copper and gallium nitrate in water with pH adjusted by adding potassium hydroxide. They are able to achieve delafossite CuGaO_2_ phase in a temperature range of 170–240 °C [34]. Tsay et al. utilized the spin coating technique, where they prepared the precursor solution by dissolving copper acetate monohydrate and gallium nitrate hydrate in 2-methoxyethanol. Subsequently, the precursor underwent spin coating at a rate of 1500 rpm for 30 s and was annealed at 900 °C for 1 h [36]. The pulsed laser deposition process conducted by Ueda et al. uses a Cu_2_O and Ga_2_O_3_ powder-pressed target. The target is eroded using a KrF excimer laser with a laser frequency of 20 Hz [37].

RF magnetron sputtering provides the flexibility to choose target materials across a wide spectrum of melting points. It also gives us the freedom to control the ratio of the material by adjusting the sputtering power of different targets. Furthermore, the RF magnetron sputtering technique gives excellent adhesion across a large surface area without requiring the use of hazardous or specialized precursors, which are necessary for CVD [38]. Delafossite CuGaO_2_ can be obtained by various sputtering methods like reactive sputtering of Cu/Ga target with O_2_ gas [39], CuO/Ga_2_O_3_ single target sputtering [27], and dual sputtering using Cu and Ga_2_O_3_ targets [8].

In this work, CuGaO_2_ thin films were deposited for the first time using the RF sputtering technique with Cu_2_O and Ga_2_O_3_ targets. This technique allows the freedom to select various sputtering power levels for the two targets to achieve the right composition. The sputtering power of the Cu_2_O was kept constant, and the Ga_2_O_3_ sputtering power was varied. The films were deposited at room temperature and then annealed in a N_2_ atmosphere. The process was optimized to obtain the delafossite phase. The structural, morphological, and optical properties of the synthesized CuGaO_2_ thin films were then studied.

## 2. Experimental

### 2.1. Deposition of CuGaO_2_ Thin Films

The CuGaO_2_ thin films used in this work were deposited using an AJA international ultra-high vacuum three-gun sputtering system. The dual targets used were 3-inch diameter of Cu_2_O (99.99% purity, Maideli Advanced Materials Co., Ltd., Jiangyin, China) and Ga_2_O_3_ (99.99% purity, Maideli Advanced Materials Co., Ltd., Jiangyin, China). The frequency of the RF magnetron sources for both targets was of 13.56 MHz. Fused quartz slides were used as substrates for depositing the films. The substrates were cleaned using acetone, methanol, and deionized water and blow-dried using nitrogen gas before the deposition. A base pressure of 5 × 10^−7^ Torr was achieved before starting the deposition. Ultra-high purity argon gas at a flow rate of 10 sccm was used as the sputtering gas. The power applied to the Cu_2_O target was kept constant at 50 W, while the power applied to the Ga_2_O_3_ target was varied between 150 and 200 W. Based on the deposition rates at different power levels, the thicknesses of the films maintained were around 2000 Å. To ensure a uniform film thickness, the substrate holder was rotated at a speed of 20 rpm. Post-deposition annealing of the films were carried out in a tube furnace at 850 °C and 900 °C. The annealing was performed for 5 h in the presence of ultra-high-purity N_2_ ambiance at a constant flow of 300 sccm. Table 1 lists the deposition parameters that were used for this work. All the data presented were verified by preparing several samples under identical conditions.

### 2.2. Film Characterization

The thicknesses of the deposited films were measured using a Veeco Dektak 150 surface profilometer (Veeco, Plainview, NY, USA). XRD analysis was performed using the PANalytical Empyrean XRD system (Malvern Panalytical, Westborough, MA, USA), using a Cu radiation source at 45 kV and 40 mA. The diffraction patterns were recorded at 2θ angles of 25°–70°. HighScore Plus software version 4.5 (Malvern Panalytical, Westborough, MA, USA) was used to analyze the phase information. The film’s composition was analyzed using ESCALAB 250 Xi + X-ray photoelectron spectroscopy (XPS) (ThermoFisher Scientific, Waltham, MA, USA) with a monochromatic source Al Kα source (1486.7 eV). Before XPS measurements, an inbuild EX06 ion source was used to perform ion milling on the sample to remove the surface oxygen. The XPS data were then analyzed using Thermo Fischer Scientific Avantage software (version 5.9902) to perform XPS peak fitting. Surface morphological studies of the films were performed using the Zeiss Ultra-55 SEM (Zeiss Microscopy, White Plains, NY, USA). Optical transmission analysis was performed at light wavelengths ranging from 300 to 800 nm using a Cary 100 UV-Vis spectrometer (Varian Analytical Instruments, Walnut Creek, CA, USA). The Tauc plot method was used to calculate the bandgap of the films. The conductivity type of the post-deposition-annealed film was found using the hot probe method.

## 3. Results and Discussion

### 3.1. XRD and XPS Analysis

**Figure 1:**
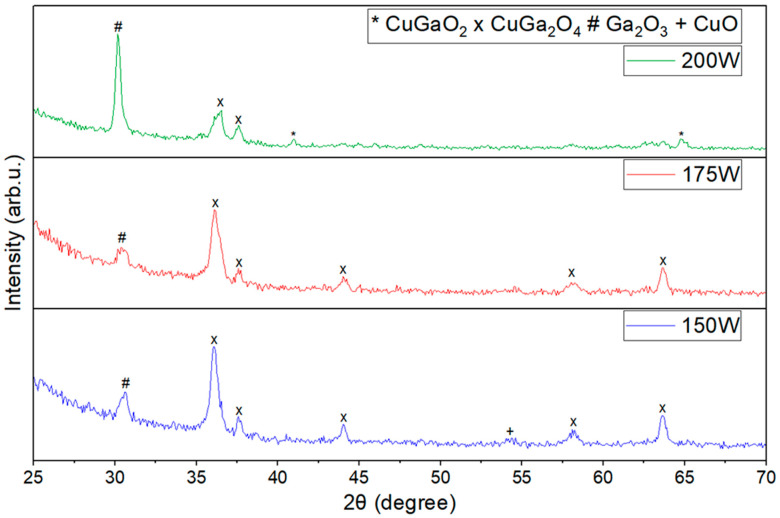
XRD patterns of films deposited at various Ga_2_O_3_ sputtering powers and annealed at 850 °C.

The XRD diffractograms of films deposited at various Ga_2_O_3_ sputtering powers (150 W, 175 W, and 200 W) and subjected to annealing at 850 °C are shown in Figure 1. The 2θ range was adjusted to eliminate the quartz amorphous peak that was observed in all films at the range between 18° and 25°. The as-deposited film did not show any diffraction peaks and confirmed to be amorphous in nature. However, all the annealed films were found to be nanocrystalline due to their distinct diffraction peaks. The lack of discernible peaks in the as-deposited films can be explained by the insufficient energy present during the deposition process, hindering crystallization. Similar to the research reported by Dong L et al. [40], distinct Ga_2_O_3_ peaks were identified at 30.22° on all the annealed thin films. Figure 1 shows the XRD diffractograms of the aforementioned films were annealed at 850 °C. As observed in Figure 1, films deposited using a sputtering power of 150 W for Ga_2_O_3_ exhibited peaks associated with spinel CuGa_2_O_4_, alongside a CuO peak detected at 54.3°. By increasing the sputtering power to 175 W, the CuO peak was no longer visible, and the remaining CuGa_2_O_4_ peaks became stronger. However, by increasing the Ga_2_O_3_ sputtering power to 200 W, peaks pertaining to CuGa_2_O_4_ started to disappear, while peaks pertaining to CuGaO_2_ started to appear. The films mentioned above were further not characterized due to the absence of CuGaO_2_ phase films.

**Figure 2:**
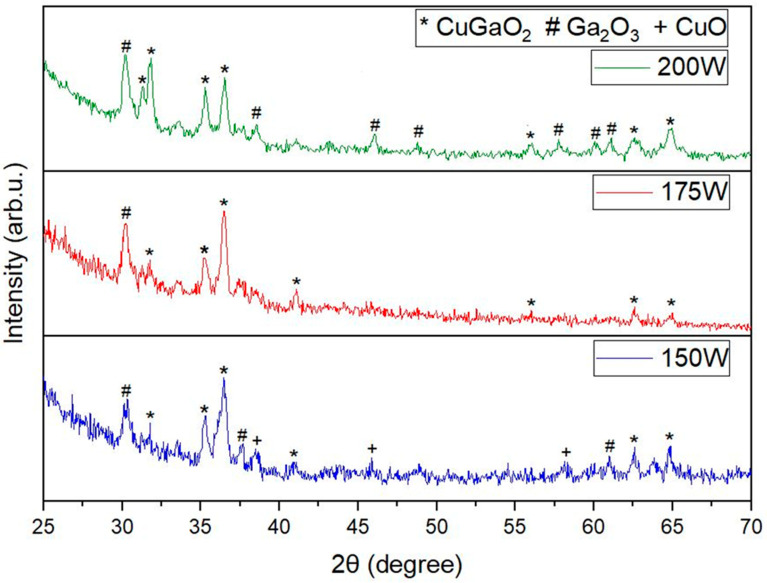
XRD patters of films deposited at various Ga_2_O_3_ sputtering powers and annealed at 900 °C.

Figure 2 shows XRD results of films annealed at 900 °C. Raising the annealing temperature to 900 °C resulted in the disappearance of peaks associated with CuGa_2_O_4_ while peaks pertaining to CuGaO_2_ emerged. Similar results were reported in earlier studies [27,41]. The films deposited at 150 W Ga_2_O_3_ power showed a combination of CuO, Ga_2_O_3_, and CuGaO_2_ peaks. With the increase in Ga_2_O_3_ sputtering power to 175 W, the peaks pertaining to CuO disappeared. This phenomenon can be attributed to the rise in Ga_2_O_3_ concentration within the film as the sputtering power for Ga_2_O_3_ increases, potentially facilitating its reaction with CuO to yield CuGaO_2_. With the exception of Ga_2_O_3_ peak at 30.22°, nearly single-phase CuGaO_2_ was identified. However, when the Ga_2_O_3_ sputtering power was increased to 200 W, the predominantly single-phase CuGaO_2_ diminished, while additional Ga_2_O_3_ peaks started emerging, attributable to the heightened Ga concentration within the film. No peaks associated with spinel CuGa_2_O_4_ were detected, when the films were annealed at 900 °C. In the film deposited with 175 W power to the Ga_2_O_3_ target, the major peaks were identified at 2θ angles of 31.4°, 35.21°, 36.43°, 41.06°, and 62.59° indexed to (006), (101), (012), (105), and (110), respectively. This shows the formation of nearly single-phase delafossite CuGaO_2_ (JCPDS PDF # 41–0255). These findings indicate that the CuGaO_2_ phase was achieved at relatively high temperatures compared to CuO and CuGa_2_O_4_ phases at low temperatures. The findings from the XRD analysis are summarized in Table 2, Table 3, Table 4, Table 5 and Table 6. Equation (1) explains the chemical reaction for the formation of CuGaO_2_ [39].

CuGa_2_O_4_ + CuO → 2CuGaO_2_ + 0.5O_2_ (1)

**Figure 3:**
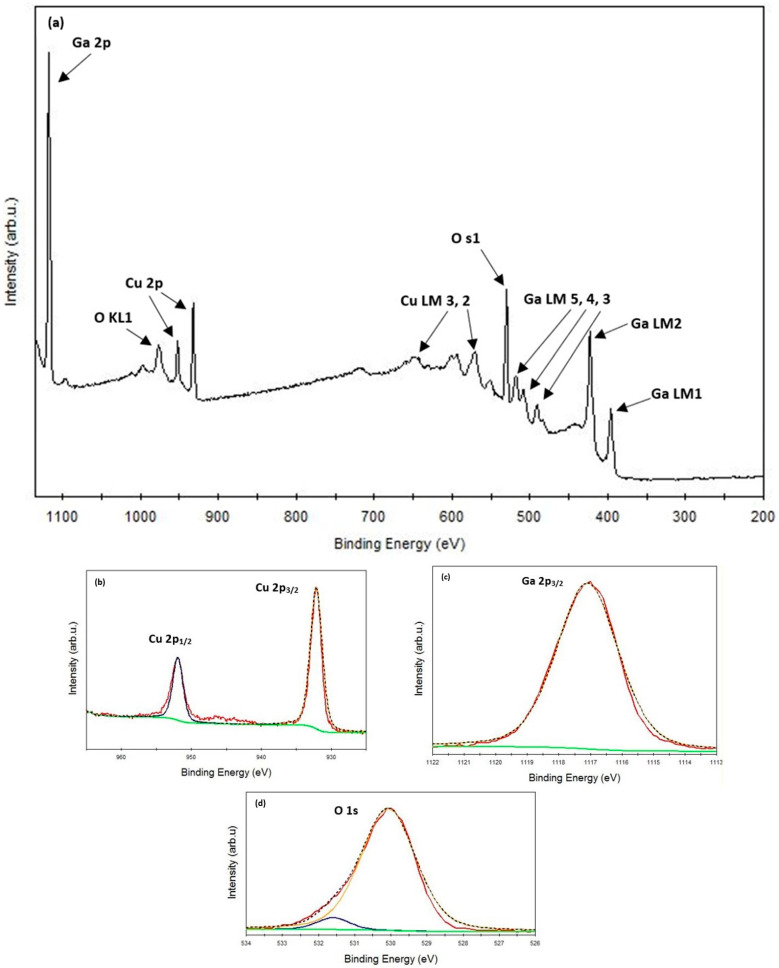
XPS spectra of the CuGaO_2_ film deposited with a Ga_2_O_3_ sputtering power set at 175 W: (**a**) survey spectrum; (**b**) Cu-2p state; (**c**) Ga-2p state; (**d**) O-1s state.

As the optimal performance was achieved by sputtering the film with a Ga_2_O_3_ sputtering power of 175 W and subsequently annealing it at 900 °C, and XPS analysis was only conducted on these films. Figure 3a shows the XPS survey spectra pertaining to the film. It was confirmed from the survey spectrum that only Cu, Ga, and O-related peaks were found. Figure 3b shows two peaks denoting Cu 2p^3/2^ and Cu 2p^1/2^ detected at binding energies of 932.13 eV and 951.93 eV, respectively. The lack of satellite peaks within the range of 940 and 950 eV served as a confirmation of the absence of Cu^2+^ species. Figure 3c shows the Ga 2p^3/2^ peak observed at 1117.09 eV. Two sub-peaks were found for the O 1s state at 530.07 eV and 531.6 eV, as seen in Figure 3d. The peak detected at a binding energy of 530.07 eV is indicative of lattice oxygen within CuGaO_2_, whereas the peak observed at 531.6 eV corresponds to chemisorbed oxygen. The peaks found at its associated binding energy are consistent with those in other reported researches [29,42].

### 3.2. Morphology Studies

**Figure 4:**
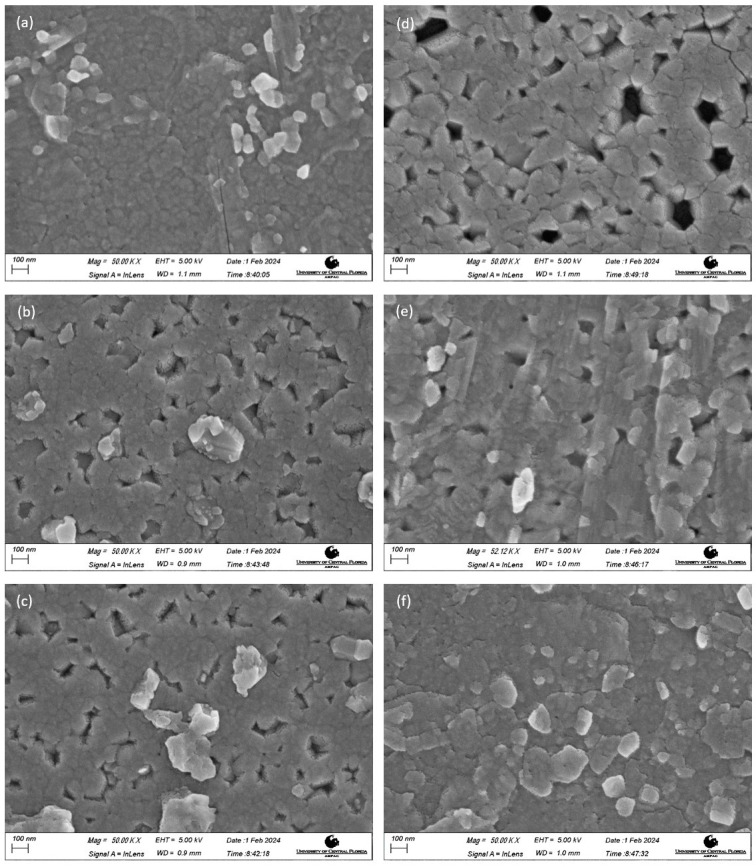
SEM images of films obtained by annealing at 850 °C with Ga_2_O_3_ sputtering powers of 150 W (**a**), 175 W (**b**), and 200 W (**c**) and annealed at 900 °C with sputtering powers of 150 W (**d**), 175 W (**e**), and 200 W (**f**).

SEM images of the post-deposition-annealed films are shown in Figure 4. All the images were obtained at a 50 K magnification. Distinct grains were seen on all the films, confirming their nano-crystallinity as previously reported in the XRD section. Figure 4a–c shows the films annealed at 850 °C, and Figure 4d–f show the films annealed at 900 °C. Overall, it was observed that an increase in annealing temperature led to an increase in the grain size of the films, as reported by [41,43].

### 3.3. Optical Studies

#### 3.3.1. Optical Transmission

**Figure 5:**
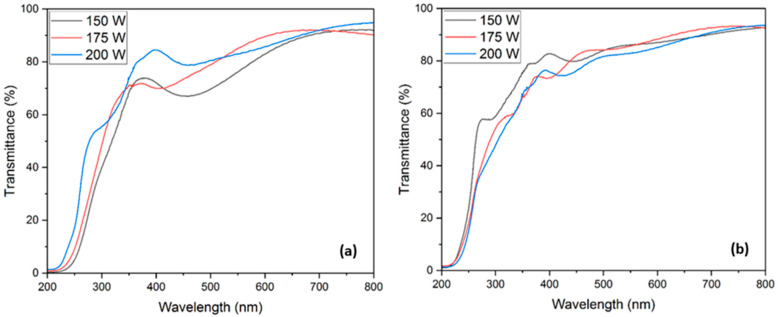
Optical transmissions of CuGaO_2_ films annealed at 850 °C (**a**) and 900 °C (**b**).

Optical transmission analyses were performed on all films deposited onto quartz slides following annealing. UV-Vis spectrophotometry recorded transmission data ranging from 200 to 800 nm. Figure 5 illustrates the optical transmissions of films post-deposition-annealed at 850 °C and 900 °C. Films annealed at 850 °C displayed a rising trend in optical transmission with the increasing sputtering power. The films deposited with Ga_2_O_3_ sputtering powers of 150 W, 175 W, and 200 W had optical transmissions of 77.15%, 82.45%, and 84.14%, respectively. However, films subjected to annealing at 900 °C exhibited a decrease in transmission, as the sputtering power increased. The films deposited with Ga_2_O_3_ sputtering powers of 150 W, 175 W, and 200 W had optical transmissions of 85.03%, 84.97%, and 82.37%, respectively. The increase and the decrease in the optical transmission perfectly aligned with the grain size change reported in the morphology studies section. As the grain size decreased, the optical transmission was identified to increase in the films annealed at 850 °C. On the contrary, the films annealed at 900 °C showed an opposite trend of reduction in optical transmission with an increase in grain size. From SEM data, we could conclude that with an increase in grain size, the optical transmission decreased. A similar correlation between optical transmission and grain size has been reported in [44,45].

#### 3.3.2. Optical Bandgap

The optical bandgap of CuGaO_2_ thin films subjected to post-deposition annealing, was determined using the Tauc plot method [46]. The absorption coefficient (α) was calculated based on the transmission spectra data using the equation:

(2) $$\alpha =\left(\frac{-2.303}{t}\right)\log_{10}\left(\%T\right)$$

where t represents the thickness of the CuGaO_2_ thin film, and T denotes the transmission of the film.

The Tauc equation was used to find the bandgap based on the absorption coefficient:

(3) $${\left(\alpha h\nu \right)}^{\left(\frac{1}{n}\right)}=B(h\nu -Eg)$$

where “*n*” indicates the nature of sample transition, “h” is the photon energy, “ν” is the vibration frequency, “hν” is the Planck’s constant, Eg denotes the optical bandgap, and B is constant. The values of *n* equal ½ for direct allowed, 2 for indirect allowed, and 3/2 for direct forbidden transitions [46,47]. Since *n* = ½ yielded the best linear fit of the ${\left(\alpha h\nu \right)}^{\left(\frac{1}{n}\right)}$ vs. the photon energy curve, the films deposited in this study show a direct bandgap transition. This is also backed by previous research on this material [37,39]. The bandgap values were determined by extrapolating the linear portion of the curves to the x-axis.

**Figure 6:**
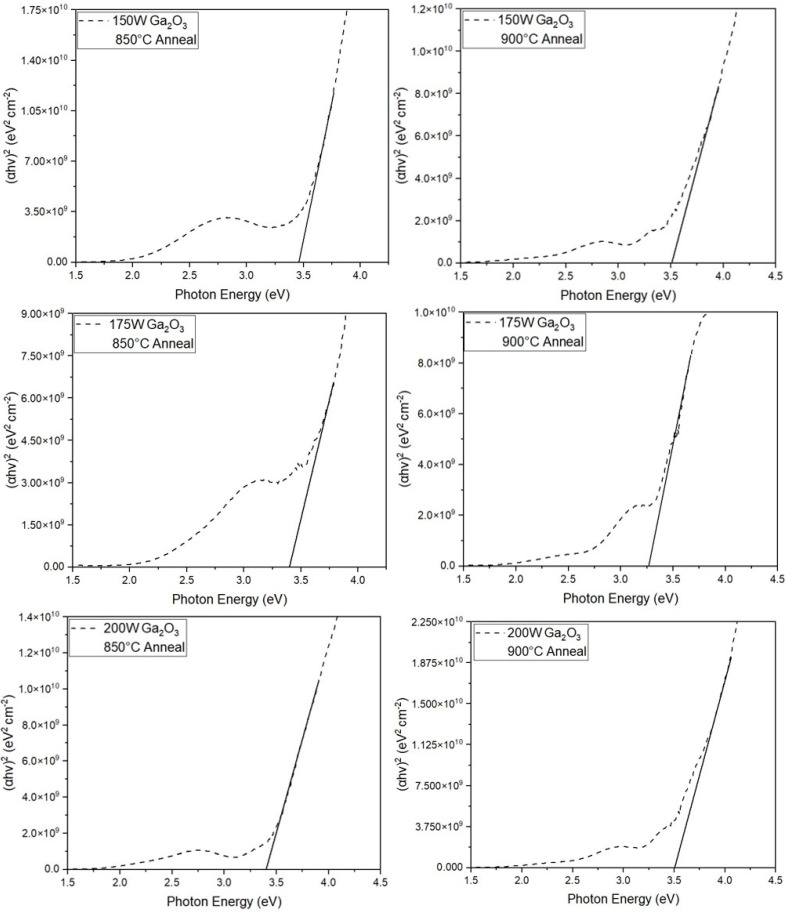
Tauc plots of CuGaO_2_ films annealed at 850 °C and 900 °C.

Figure 6 illustrates the Tauc plots for films subjected to annealing temperatures of 850 °C and 900 °C. For films deposited using Ga_2_O_3_ sputtering powers of 150 W, 175 W, and 200 W and annealed at 850 °C, the corresponding optical bandgaps were measured to be 3.45 eV, 3.39 eV, and 3.4 eV, respectively. Similarly, films deposited under the same power settings and annealed at 900 °C exhibited optical bandgaps of 3.54 eV, 3.27 eV, and 3.5 eV, respectively. As seen in the XRD results in Figure 2, the films deposited with Ga_2_O_3_ powers of 150 W and 200 W had a higher Ga_2_O_3_ content, which is known to have a higher bandgap than CuGaO_2_. The decrease in the bandgap of the 175 W film can be attributed to the fact that it was nearly single-phase CuGaO_2_, which closely matches other reported results [48]. Copper-based delafossites like CuGaO_2_, CuCrO_2_, and CuInO_2_ are known to exhibit p-type conductivity owing to its intrinsic defects such as interstitial oxygen ions and/or Cu vacancies in the copper lattice [49,50]. This was further verified using the hot probe test on all the films showing p-type behavior.

#### 3.3.3. Refractive Index Studies of CuGaO_2_

The refractive index of the nearly single-phase CuGaO_2_ thin film was determined utilizing the following formula [51]:

(4) $$\frac{1}{2\eta t}=\frac{1}{\lambda_{m+1}}-\frac{1}{\lambda_{m}}$$

where η represents the refractive index of the CuGaO_2_ film, t indicates the thickness of the film, and λ*_m_*_+1_ and λ*_m_* denote the wavelengths where the successive maxima are observed in the transmission spectrum. The refractive index of the CuGaO_2_ thin film was found to be 2.5 in the 300–450 nm wavelength range which is similar to the previously reported data [52].

## 4. Conclusions

In this study, thin films of CuGaO_2_ were successfully deposited through a dual sputtering method utilizing Cu_2_O and Ga_2_O_3_ targets. These films were deposited on a quartz substrate, maintaining the Cu_2_O sputtering power at a constant 50 W while varying the Ga_2_O_3_ power. Subsequently, the samples underwent annealing at temperatures of 850 °C and 900 °C in a N_2_ atmosphere, followed by analysis of their structural and optical characteristics using XRD, XPS, SEM, and UV-Vis spectroscopy. When annealed at 900 °C, the films deposited at a Ga_2_O_3_ sputtering power of 150 W showed the presence of CuO, and the films deposited with a sputtering power of 200 W were Ga_2_O_3_ phase-rich. Nearly single-phase delafossite CuGaO_2_ films were obtained with a Ga_2_O_3_ sputtering power of 175 W. CuGa_2_O_4_ peaks were observed, when the film was annealed at 850 °C. SEM images revealed an increasing trend in grain size when annealed at 850 °C and a decrease in grain size when annealed at 900 °C. The optical transmission of the film increased with the decrease in grain size. The nearly single-phase CuGaO_2_ film had an optical transmission of about 85% at the visible range, and its optical bandgap was found to be 3.27 eV. The refractive index of the film was found to be 2.5 in the 300–450 nm wavelength range.

## Figures and Tables

**Table 1:** Deposition parameters maintained for the work.

Deposition Parameter	Specification
Base pressure	5 × 10^−7^ Torr
Deposition pressure	10 mTorr
Sputtering gas	Ar
Sputtering gas flow rate	10 sccm
Cu_2_O power	50 W
Ga_2_O_3_ power	150 W, 175 W, and 200 W
Substrate temperature	Room temperature
Thickness of the deposited film	200 nm
Annealing temperature	850 °C and 900 °C
Annealing time	5 h
Annealing gas and flow rate	N_2_ at 300 sccm

**Table 2:** Summary of the peaks identified for the various deposition parameters.

	850 °C	900 °C
150 W	CuGa_2_O_4_ + Ga_2_O_3_ + CuO	CuGaO_2_ + Ga_2_O_3_ + CuO
175 W	CuGa_2_O_4_ + Ga_2_O_3_	CuGaO_2_ + Ga_2_O_3_
200 W	CuGa_2_O_4_ + CuGaO_2_ + Ga_2_O_3_	CuGaO_2_ + Ga_2_O_3_

**Table 3:** Major peak indices of CuGaO_2_.

2θ Angle	Index
31.4°	(006)
35.21°	(101)
36.43°	(012)
41.06°	(105)
62.59°	(110)

**Table 4:** Major peak indices of CuO.

2θ Angle	Index
38.69°	(111)
45.09°	$(11\bar{2}$)
57.94°	(202)

**Table 5:** Major peak indices of Ga_2_O_3_.

2θ Angle	Index
30.22°	(400)
38.55°	(401)
46.05°	(202)
48.98	$(\bar{5}$01)
57.8	(511)
59.97	$(\bar{8}$01)

**Table 6:** Major peak indices of CuGa_2_O_4_.

2θ Angle	Index
36.17°	(311)
37.71°	(222)
44.08°	(400)
58.29°	(333)
63.68°	(440)

## Data Availability

Data are contained within the article.
